# The Effects of Spironolactone in Preventing Bile Duct Ligation-induced Hepatitis in A Rat Model

**DOI:** 10.22037/ijpr.2020.112488.13786

**Published:** 2021

**Authors:** Ahmet Özer Şehirli, Azime Kökeş, Ayliz Velioğlu-Öğünç, Şermin Tetik, Naziye Özkan, Şule Çetinel, Serkan Sayıner, Gül Dülger

**Affiliations:** a *Department of Pharmacology, Faculty of Dentistry, Near East University, 99138 Nicosia, North Cyprus, Mersin 10, Turkey. *; b *Department of Pharmacology, Faculty of Pharmacy, Marmara University, 34722 Istanbul, Turkey. *; c *Vocational School of Health-Related Professions, Marmara University, 34722 Istanbul, Turkey.*; d *Department of Biochemistry, Faculty of Pharmacy, Marmara University, 34722 Istanbul, Turkey. *; e *Department of Histology and Embryology, School of Medicine, Marmara University, 34722 Istanbul, Turkey. *; f *Department of Biochemistry, Faculty of Veterinary Medicine, Near East University, 99138 Nicosia, North Cyprus, Mersin 10, Turkey.*

**Keywords:** Cholestasis, Inflammation, Multidrug resistance-associated protein 2, Pregnane X receptor, NF-ĸB, Spironolactone

## Abstract

Cholestasis is associated with the accumulation of bile acids and bilirubin in the hepatocytes and leads to liver injury. Pregnane X Receptor (PXR) coordinates protective hepatic responses to toxic stimuli, and this receptor was reported to stimulate bile secretion by increasing MRP2 expression. Since PXR activators were reported to be anti-inflammatory in the liver, PXR was proposed as a drug target for the treatment of chronic inflammatory liver diseases. We investigated the potential protective effect of spironolactone (SPL), an enzyme inducer, in hepatotoxicity induced by bile duct ligation in rats. Wistar Albino (250-300 g) rats were divided into the control group and the bile duct ligated (BDL) group. BDL group was divided into three subgroups; following BDL, for 3 days, the first group received propylene glycol (vehicle of SPL) (blinded), the second subgroup received spironolactone (SPL) (200 mg/kg oral), and the third subgroup received SPL for 3 days, starting 3 days after the bile duct ligation, in order to investigate if it has a healing effect after hepatitis had developed. The control group was sham-operated and received saline. At the end of the experiment, blood and tissue samples were collected. Serum TNF-α, NF-ĸB, bilirubin, IL-6 levels, ALT, AST, ALP activities and tissue MPO activity and oxidant damage increased after the bile duct ligation was significantly decreased following SPL administration. PXR and MRP2 activity showed an increase in the hepatocytes as a result of the treatment. In conclusion, it was observed that SPL administration significantly decreases liver inflammation and damage related to BDL.

## Introduction

Chronic cholestatic liver diseases are characterized by defective bile acid transport from the liver to the intestine, caused by primary damage to the biliary epithelium. Bile acid accumulation in the liver is implicated as the principal cause of hepatotoxicity, leading to reactive oxygen species formation and oxidative damage (1). 

Disorders in the accumulation and secretion of bile acids and bile salts in the liver are often reported to be caused by down-regulation in systems related to hepatic efflux transporters such as multidrug resistance-associated protein 2 (MRP2) (1–4). MRPs are members of the ABC superfamily of adenosine triphosphate-dependent transporters and are expressed in various tissues, including the intestine, liver and kidneys. They facilitate the movement of various compounds, including bile acids, out of the hepatocytes. Although the molecular mechanisms related to this down-regulation phenomenon have not been fully elucidated, it is known that the activation of nuclear proteins such as pregnane X receptor (PXR), which is responsible for the increase in expression of genes related to liver detoxification and elimination of xenobiotics and endobiotics in the liver, is suppressed. On the contrary, it was reported that PXR activation is anti-inflammatory in the liver and that it causes inhibition of NF-κB activation, as well as reducing the expression of inflammatory cytokines such as TNF-α (5, 6). 

Spironolactone (SPL) is a diuretic known for its potent enzyme induction activity. It is widely used as a diuretic in patients with edema or ascites. Previous studies have shown that it induces MRP2 in the small intestine and liver of rats (4, 7 and 8). Ethinylestradiol-induced cholestasis has also been reported to be reduced by SPL, possibly through the induction of MRP2 (9).

Since SPL was reported to increase bile flow, and since it is widely used in the treatment of cirrhosis, in this study, we aimed to investigate whether or not SPL, besides being a diuretic, has any other preventive effect in BDL-induced hepatitis development in rats, and whether induction of PXR and/or MRP2 plays a part in this effect 

## Experimental

*Animals*

Albino Wistar rats of both sexes weighing between 250-300 g were acclimatized to laboratory conditions (+20 °C ± 2, 12 h light-dark cycle) for two weeks prior to the experiment, with free access to standard pellet chow and water. With respect to the laboratory analyst and statistical analyst, they were blinded to the groups and treatments allocated to the rats. This study was conducted with the consent of the Animal Ethics Committee of the Marmara University (Approval no: 45.2010 mar). 

*Surgery and Experimental Design*

Four groups of 6 rats were formed in our study. All animals were weighed at the beginning of the experiment. They were then divided into the control group, the BDL group, the SPL1 group and the SPL2 group. All rats were anesthetized (ketamine and 0.75 mg/kg chlorpromazine, i.p.), and the abdomen was opened via a midline laparotomy. In the control group (sham-operated), the rats were incised, the incision was resealed, and only saline (SF) was administered. For the induction of cholestasis (BDL group), the bile duct was doubly ligated at the hepatic hilum. This group was divided into three subgroups; the rats in the SPL1 group received spironolactone (Aldactone tablets, Ali Raif Pharmaceuticals, Istanbul, Turkey) (200 µmol/kg oral, dissolved in 60 mM propylene glycol (10)) daily for 3 days, starting on the day of the operation. The rats in the SPL2 group received SPL (200 µmol/kg oral, dissolved in 60 mM propylene glycol) daily for three days, starting 3 days after the operation. The BDL group received 60 mM propylene glycol oral (the vehicle) for 3 days. At the end of the experiment, 18 h after the last dose, the animals were decapitated and blood and tissue (liver) samples were collected. Tissue samples were stored in formalin for histological examination and at -80 °C for biochemical evaluations.

*Liver function tests*

As the markers of hepatic function and tissue injury, the activities of alanine aminotransferase (ALT), aspartate aminotransferase (AST) and alkaline phosphatase (ALP) enzymes, and the total and direct bilirubin (TBIL, DBIL) concentrations were studied with an automated analyzer (Bayer OpeRA, Bayer Corp., Germany) using routine analysis kits (respectively REF 80027, REF 92025, REF80014, REF 80403, Biolabo Europe S.A., Maizy, France).

*Measurement of Serum Cytokines Concentrations*

Serum NF-ĸB, TNF-α and IL-6 concentrations quantified according to manufacturer's instructions and guidelines using rat-specific commercially available ELISA test kits (BioSource Europe S.A.; Nivelles, Belgium). 

*Determination of tissue Myeloperoxidase (MPO) activity*

Determination of the myeloperoxidase (MPO) level in the tissue was made by the Hillegeas method (11). Immediately after decapitation, the extracted tissues were washed with saline to remove blood and debris, then dried with filter paper and weighed. The tissue samples were homogenized in 10 vol of ice-cold potassium phosphate buffer (50 mM/L K2HPO4 (pH 6) containing hexadecyltrimethylammonium bromide (HETAB; 0.5%, w/v). The homogenate was centrifuged at 30,000g for 10 min at 4 °C, and the supernatant was discarded. The pellet was then rehomogenized with an equivalent volume of 50 mmol//L K2HPO4 containing 0.5% (w/v) HETAB (Sigma-Aldrich, St. Louis, USA) and 10 mmol/L ethylenediaminetetraaceticacid (EDTA, Sigma). MPO activity was assessed by measuring the H2O2-dependent oxidation of o-dianisidine dihydrochloride (Sigma-Aldrich). One unit of enzyme activity was defined as the amount of the MPO present per gram of tissue weight that causes a change in absorbance of 1.0/min at 460 nm and 37 °C. 

*Chemiluminescence Experiment*

The chemiluminescence (CL) of luminol and lucigenin was detected to measure reactive oxygen species in tissues. Measurements were made at room temperature using a Junior LB 9509 luminometer (EG&G Berthold, Germany). The samples were placed into vials containing PBS-HEPES buffer (20 mM HEPES, pH 7.2, 0.5 M PBS). Reactive oxygen species were measured after adding enhancers lucigenin or luminol at 0.2 mM final concentration. Luminol detects reactive species; namely, it is selective for -OH, H2O2, HOCl radicals, and lucigenin is selective for O−2 (12, 13). Measurements were made at one-minute intervals and results were given as the area under the curve for a measurement duration of 5 min. The results were corrected for wet tissue weight (relative light units (rlu)/mg tissue) (14).

*Histopathological Examination*

The tissues were washed in tap water for at least 3 h or 1 night after being taken into 10% formalin, and dehydration was performed with increasing alcohol concentrations (15 min with 70% alcohol, 15 min with 90% alcohol, 30 min with 96% alcohol, twice for 30 min with 100% alcohol, and twice for 30 min with 100% toluene). Subsequently, they were kept in paraffin blocks at 60 °C for 1 night and the next day, the tissue was embedded in paraffin blocks. After blocking, sections of 5-6 mm thickness were obtained from the tissues and placed on the slide and left in toluene for 2 h for separating from paraffin, then reduced to water with reduced concentrations of alcohol (2 min with 100% alcohol, 2 min with 90% alcohol, 2 min with 70% alcohol) and left in distilled water, treated with hematoxylin for 15 min, then left for purpling for 10 min in tap water. After applying distilled water with eosin for another 5 min, dehydration was carried out with increasing alcohol concentrations (2 min with 70% alcohol, 2 min with 90% alcohol, 2 min with 96% alcohol, 10 min with 100% alcohol). After this it was washed 2 times with toluene (1st bath for 5 min, 2nd bath for 10 min) and covered with the Entellan before examining under the light microscope.

*Immunohistochemistry*

The Streptavidin-Biotin peroxidase immunohistochemistry staining method was performed to demonstrate immunoexpression of MRP2 and PXR in formalin-fixed paraffin-embedded tissues. In this method, 3μm thick sections were obtained from the paraffin-embedded tissues to positively charged slides and deparaffinized at 37 ºC for 1 night, followed by deparaffinization by keeping in three separate xylenes for 5 min. The sections were passed through two separate 96% ethanol for 10 min, and the endogenous peroxidase activity in the tissue was suppressed with 3% hydrogen peroxide (in methanol) for 20 min. The sections that were washed with distilled water were subjected to an antigen recovery process with a 200W pH 6 citrate buffer solution in a microwave oven to expose the masked antigens. The slides cooled to room temperature was washed with two separate phosphate buffer solutions (PBS) for 5 min and 10 min of protein blocking (Histostain Bulk Kit, Invitrogen LAB-SA Detection System, UK) was carried out to prevent non-specific staining. Following blocking, MRP2 (M2 III-6, Abcam, UK) dilution of 1:100, and PXR (bs-2334R, Bioss, USA) at a dilution of 1:200 was instilled and the specimens were incubated at room temperature for 1 h. This was followed by washing the sections with two separate PBS for 5 min each, and they were placed in biotinylated secondary antibody (Histostain Bulk Kit, Invitrogen LAB-SA Detection System, UK) for 10 min. After washing again with PBS, streptavidin peroxidase (Histostain Bulk Kit, Invitrogen LAB-SA Detection System, UK) was instilled, and they were incubated for 10 min. 3,3’-diaminobenzidine (DAB) chromogen was instilled into the sections that were washed with two separate PBS for five min each, and staining was checked after 5 min of incubation. The sections washed with distilled water were counterstained with Mayer's hematoxylin and dehydrated by passing through ethanol. Tissues that were transferred into xylene and covered with appropriate closure material were evaluated semi-quantitatively with a light microscope (BX51 Olympos, Japan). 

*Chemicals and reagents*

Chemicals and reagents used for the study included SPL (Aldactone tablets, Ali Raif Pharmaceuticals, Istanbul, Turkey), ELISA kits specific for rat cytokine NF-κB (Biosource International, Nivelles, Belgium), HETAB (Sigma-Aldrich, St. Louis, USA), o-dianisidine 2HCl (Sigma-Aldrich), lucigenin (bis-N-methylacridiniumnitrate), and luminol (5-amino-2,3-dihydro-1,4-phthalazinedione (Sigma-Aldrich). Chemicals and reagents used for immunohistochemistry were a serum-blocking solution (Histostain Bulk Kit, Invitrogen LAB-SA Detection System, Paisley, UK) and antibodies to PXR (bs-2334R, Bioss, Salem MA, USA) Mrp2 (M2 III-6, Abcam, UK). All other chemicals used in this study were analytical grade.

*Statistical analysis*

Statistical analyses were performed using the GraphPad Prism 6.0 (GraphPad Software, San Diego, CA, USA). All findings were expressed as mean ± standard deviation. Statistical analysis was performed with the one-way analysis of variance (ANOVA) and Tukey's test for further analysis. P values lower than 0.05 were considered significant.

## Results

It was observed that ALT, AST, ALP, total bilirubin and direct bilirubin levels were significantly increased in the saline-treated BDL rats compared to the sham-operated control group (*p* < 0.001). The increase in AST, ALT, ALP, total bilirubin and direct bilirubin levels were suppressed significantly in both (SPL1) (*p* < 0.05) and (SPL2) (*p* < 0.001) groups (Table 1). The suppression appeared to be more prominent in the SPL2 group (*p* < 0.001) compared to the SPL1 group (*p* < 0.05). However, the difference between the two groups was not found to be significant, except for the ALP group (*p* < 0.05) (Table 1). 

The serum levels of NF-ĸB, TNF-α and IL-6 are indicators of inflammation in the cirrhosis and they were increased significantly in the BDL group compared to the control group (*p* < 0.01). In the treatment groups, the increased NF-ĸB and TNF-α levels were suppressed significantly (*p* < 0.01). The decrease in the NF-ĸB level was more significant in the SPL1 group than in the SPL2 group. Although there was suppression in the increase in IL-6 levels in groups treated with SPL, no significant decrease was observed (Table 2).

The hepatic MPO activity, luminol and lucigenin levels in the liver tissue were significantly increased in the BDL group compared to the control group (*p* < 0.001). In the treatment groups, MPO activity was decreased significantly, but again, there was no significant difference between the SPL1 and SPL2 groups. Luminol and lucigenin activity of the tissue are indictors of oxidative damage. After BDL, luminol and lucigenin activities in the liver tissue were increased significantly, but SPL treatment prevented oxidative damage almost completely, bringing the values back to the control levels (Table 3). 

**Figure 1:**
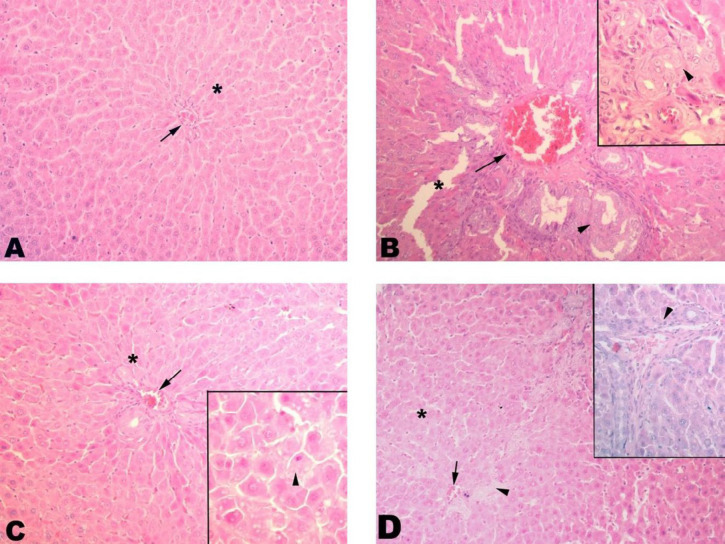
Liver histopathology. (A) Control group. Control group with proper sinusoids (*) and central vein (arrow); (B) BDL group. Significant dilatation (*) and fibrosis (arrowhead) of sinusoids are seen. Congestion in the central vein is remarkable (arrow); (C) SPL1 group. Dilatation (*) in the sinusoids and congestion in the central vein (arrowhead) regressed. The contours of the hepatocytes are smooth (arrowhead); (D) SPL2 group. Dilatation (*) and congestion (arrow) in sinusoids regressed but still low level of fibrosis (arrowhead) is seen

Histologically, control liver tissue showed a smooth structure with a central vein and sinusoids extending therefrom. The BDL group showed severe fibrosis with severe sinusoidal dilatations. Better regeneration was seen in the SPL1 group than in the SPL2 group, with improved sinusoidal structures and decreased fibrosis (Figure 1). 

**Figure 2:**
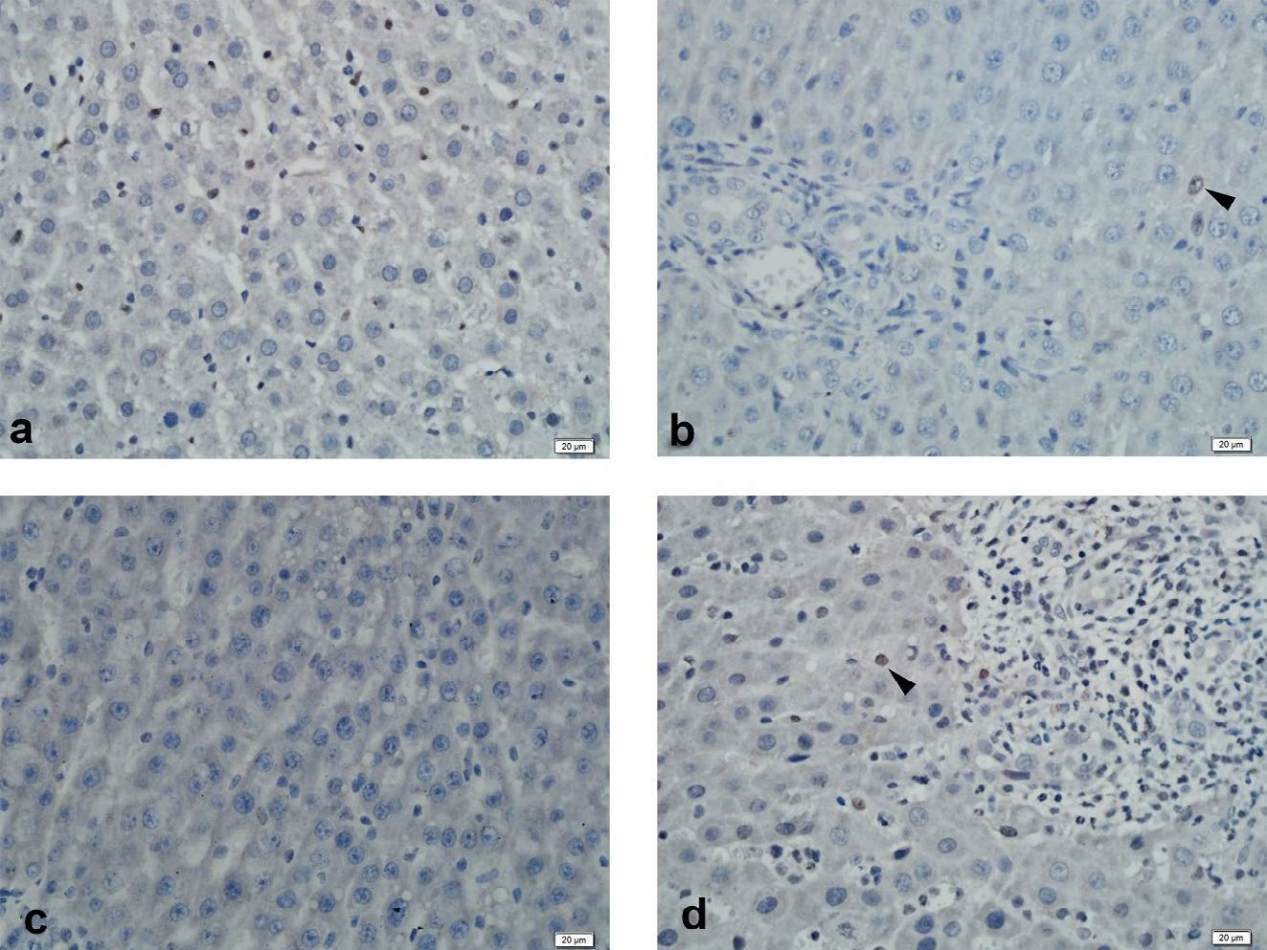
Immunohistochemistry of liver PXR. (a) Control Group: Mild staining; (b) BDL Group: Mild staining; (c) SPL1 Group: No staining; (d) SPL2 Group: Mild staining

**Figure 3:**
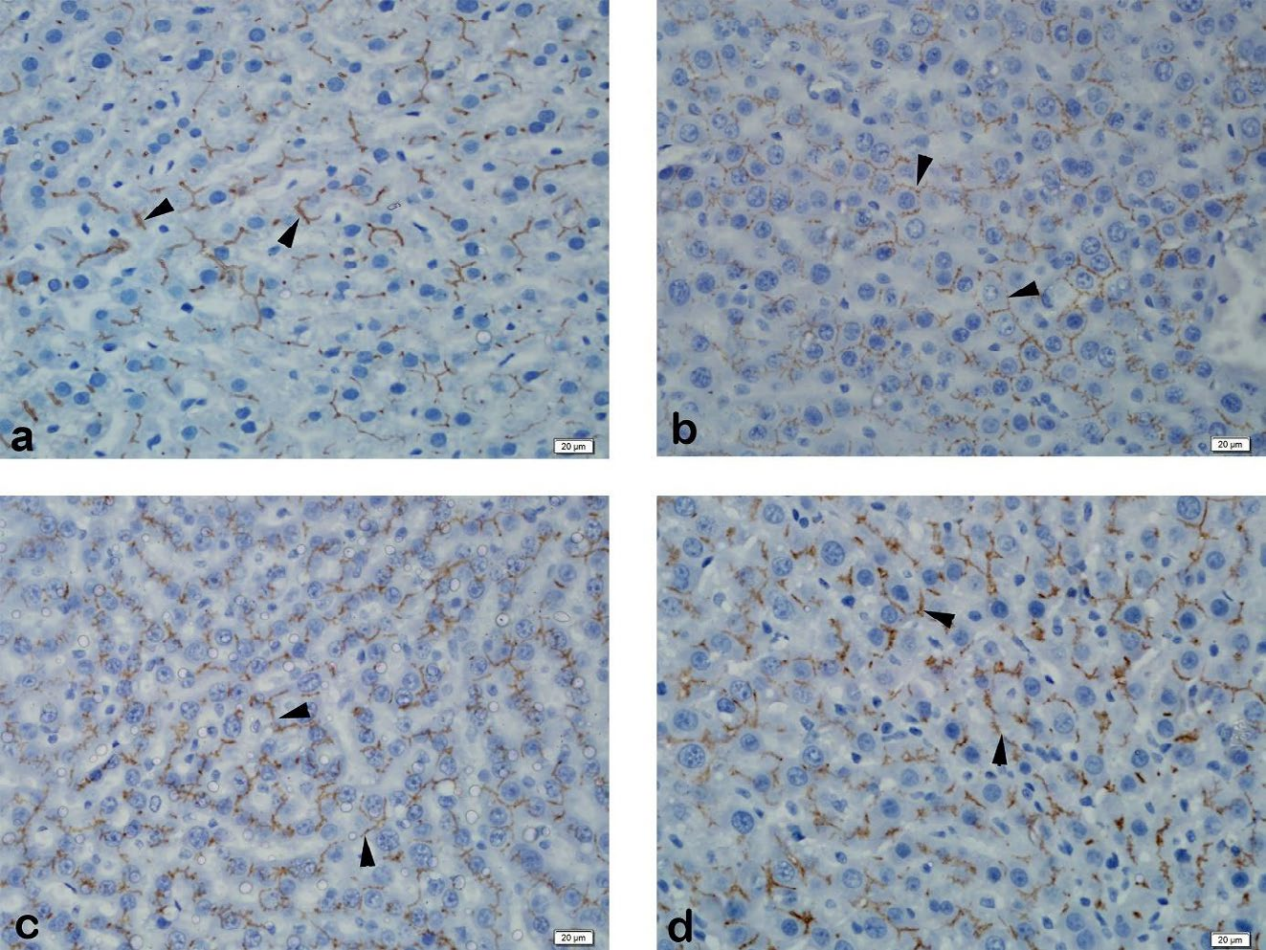
Immunohistochemistry of liver MRP2. (a) Control Group: Strong staining observed; (b) BDL Group: Strong staining observed; (c) SPL1 Group: Strong staining observed; (d) SPL2 Group: Strong staining observed

Immunohistochemical examination of MRP2 immunoexpression revealed strong staining of liver tissue in the control group. Strong liver staining was observed in the BDL group. Strong staining was observed in the SPL1 and the SPL2 groups (Figure 2). In PXR immunoexpression, liver tissue was observed to be slightly stained in the control group. In the BDL group, liver tissue showed mild staining. No staining was observed in the liver in the SPL1 group. In the SPL2 group, mild staining of the liver was observed (Figure 3).

## Discussion

Spironolactone is an aldosterone antagonist diuretic agent that also has a strong enzyme-inducing property. Spironolactone has been used as a diuretic for many years in patients with cirrhosis and has been reported to be effective in reducing edema that had accumulated in the body (7). Previously, Ruiz *et al. *(2005; 2009) reported that SPL treatment improves liver injury in ethinylestradiol-induced cholestasis by preventing the accumulation of bile acids and salts in hepatocytes via induction of MRP2 (7, 8). In the present study, hepatitis was induced by bile duct ligation, and following the operation, the rats were administered oral spironolactone for three days. Histomorphological deterioration of liver tissues in BDL rats treated with SPL returned to normal following treatment. Significant decreases in serum ALT, ALP, AST, bilirubin levels, which are indicators of liver injury, and NF-ĸB, IL-6, TNF-α cytokines, which are indicators of inflammation, were observed. These findings and also the decrease in oxidative damage indicate that SPL treatment provides an effective treatment of the liver injury induced by BDL. The immunohistochemical study results indicate that there was an increase in the expression of the transport protein MRP2 levels in hepatocytes after SPL treatment, although PXR expression did not seem to be affected.

In general, MPO activity, a neutrophil-specific enzyme, is measured to evaluate neutrophil tissue infiltration into the tissue, and studies have reported increased MPO activity due to bile duct ligation (15). Decreased MPO activity in tissues of rats treated with SPL indicates that this treatment reduces the neutrophil infiltration into the tissue, thereby reducing neutrophil-induced oxidative tissue damage (16). 

The increased reactive oxygen derivatives demonstrated by chemiluminescence in the liver tissue also support the view that free radicals play an important role in BDL-induced liver damage (17). Free oxygen radicals damage various biomolecules present in tissues, resulting in cell destruction due to the degradation of cell integrity and membrane permeability. Thus, oxygen radicals are involved in impaired cell function and liver necrosis in rats with biliary obstruction (18). In our study, as shown by the chemiluminescence measurements, oxidative damage increased significantly due to BDL, and SPL administration significantly reduced this damage.

In cholestatic liver diseases and in cases where the bile acid pool in the enterohepatic circulation is depleted, it is observed that the function of the transport proteins in liver function is impaired. As a result, organic anions such as bile salts and bilirubin glucuronides accumulate in hepatocytes. (19, 20). Regulation of MRP2expression is not well understood; it has been reported that MRP2 is downregulated in the rat model with cholestasis and that it is up-regulated in cultured rat hepatocytes exposed to cycloheximide. In their study, Tanaka et al. demonstrated an increase in serum bilirubin levels and daily urinary excretion of conjugated bilirubin after BDL. Under normal physiological conditions, the expression of MRP2 is high, and that of MRP3 is low in hepatocytes; therefore, in the normal liver, most of the conjugated organic anions are transported to bile canaliculi via MRP2. Low expression of MRP2 and concomitant increased expression of MRP3, which is located on the basolateral membrane of hepatocytes, may explain the elevation of serum bilirubin during BDL. MRP3 in the liver is reported to be up-regulated by bilirubin glucuronides (1). In a 1997 study by Trauner et al., down-regulation of MRP2 levels in the liver was observed in the bile duct-ligated rats, which resulted in elevated serum total bilirubin and serum ALP levels (21). 

In parallel with these findings, in our study, the serum bilirubin levels increased significantly after BDL, which may result from down-regulation of MDR2 expression, and this increase was prevented significantly in the groups treated with SPL, probably due to induction of MRP2 expression. It may be proposed that the reason for the decrease in serum bilirubin levels is the decrease in the rate of bile acid excreted into the blood due to the MRP2 induction in hepatocytes (8). 

Hepatobiliary transport proteins are down-regulated in toxic or cholestatic liver injury. Various pro-inflammatory cytokines such as TNF-α and IL-6, which are activated in cholestasis, are responsible for this regulation. In-vitro and in-vivo studies have shown that excretion of bile components and uptake is decreased in inflammation with increased cytokines such as TNF-α and IL-6 (22, 23). NF-ĸB is the main transcriptional regulator of immune and inflammatory responses, and TNF-α is a pro-inflammatory cytokine that viral conditions can activate, reactive oxygen species and UV rays. Increased NF-ĸB also increases the expression of TNF-α. Thus, when NF-ĸB is suppressed, TNF-α is also suppressed (23). A study by Zhou *et al*., it was shown that activation of NF-ĸB inhibits PXR activity and that inhibition of NF-ĸB increases the PXR-mediated MRP2 expression by recovering the suppressed PXR activation (24).

Similarly, activation of PXR by rifampicin, an active PXR ligand in humans, has been shown to inhibit NF-kB activation (5). In the present study, cholestasis-induced inflammation in rats undergoing bile duct ligation was shown by the significant increase in serum levels of TNF-α, NF-ĸB and IL-6 pro-inflammatory cytokines compared to the control group. Three-day SPL treatment resulted in a decrease in these parameters. SPL is known to cause potent enzyme induction and exert its effect via the PXR-activating mechanism. Suppression of pro-inflammatory cytokines by PXR activation has been shown to reduce inflammation and hepatotoxicity in many studies, as mentioned above (25, 26). 

Previous studies have demonstrated that PXR is the main regulator of inflammation, suggesting that PXR levels do not change due to metabolic reasons (27). However, in the present study, we did not observe any significant change in PXR expression, although treatment had a significant anti-inflammatory and antioxidant effect. Also, MRP2 expression was significant. It may be suggested that the reason for not demonstrating the probable change in PXR expression in the present study may be due to some methodological errors.

In SPL administrated rats, the hepatotoxicity that was caused through the accumulation of toxic bile components in the liver was reduced as a result of induction of several transport proteins, mainly MRP2, but it is also accepted that because of the potent diuretic effect of SPL, hepatotoxicity is prevented by increased excretion of bile components from the kidneys; briefly, spironolactone appears to decrease the serum bilirubin levels by increasing the elimination of bile salts through different mechanisms. 

The reason for forming two treatment groups as SPL1 and SPL2 in our study was to wait for 3 days after ligation of the bile duct and determine whether the rats responded to the treatment after hepatitis formation. Elevated AST, ALT, and ALP levels in liver function tests showed a more significant decrease in the SPL2 group than in the SPL1 group. This can be explained by the fact that, after the onset of hepatitis, before administration of SPL, the body's protective mechanisms are activated to prevent damage and reduce enzyme levels. NF-ĸB and MPO levels were higher in the SPL2 group compared to the SPL1 group. However, there was no significant difference between the two groups in the measured parameters except for the ALP levels. In light of this information, the treatment with SPL seems effective even if it is started after the onset of hepatitis.

 In conclusion, in the present study, SPL was demonstrated to reduce the inflammation and decrease the tissue damage in BDL induced hepatitis probably by regulating the expression of transport proteins in liver tissue maybe also by inhibiting the activation of inflammatory cytokine., These observations indicate that, besides its diuretic effect, spironolactone offers various other beneficial effects in the treatment of inflammatory liver diseases such as hepatitis. 

**Table 1:** Serum, AST, ALT, ALP, total bilirubin and direct bilirubin levels of all groups in cirrhosis model formed by bile duct ligation (BDL) in rats (n = 6; mean ± SD).

	**Control**	**BDL**	**SPL1**	**SPL2**
AST (U/L)	31.60 ± 5.94	1221± 256^***^	733± 192^***, ++^	612 ± 258^**, +++^
ALT (U/L)	36.60 ± 5.17	510 ± 172^***^	325 ± 94^**, +^	186 ± 48^+++^
ALP (U/L)	89.2 ± 12.7	586 ± 238^***^	411 ± 112^**^	140 ± 37^+++, γ^
Total Bilirubin (mg/dL)	0.48 ± 0.29	6.12 ± 2.43^***^	1.68 ± 0.39^+++^	2.11 ± 0.78^+++^
Direct Bilirubin (mg/dL)	0.32 ± 0.14	4.29 ± 1.89^***^	1.29 ± 0.39^+++^	1.52 ± 0.61^+++^

^*^p < 0.05, ^**^p < 0.01, ^***^p < 0.001 compared to the control group; ^+^*p <* 0.05, ^++^*p <* 0.01, ^+++^*p <* 0.001 compared to the BDL group; ^γ^*p <* 0.05 compared to SPL1 group.

**Table 2:** The serum NF-κB, TNF-α and IL-6 levels of all groups in the cirrhosis model formed by bile duct ligation (BDL) in rats (n = 6; mean ± SD).

	**Control**	**BDL**	**SPL1**	**SPL2**
NF-κB (pg/mL)	19.71 ± 13.49	52.14 ± 6.31^**^	19.22 ± 13.99^++^	41.54 ± 14.60
TNF-α (pg/mL)	839 ± 97	1158 ± 72^*^	891 ± 55 ^+^	854 ± 141^+^
IL-6 (pg/mL)	372 ± 147	657 ± 133^*^	631 ± 137	550 ± 169

^*^p < 0.05, ^**^p < 0.01 compared to the control group; ^+^*p <* 0.05, ^++^*p <* 0.01 compared to the BDL group.

**Table 3:** Tissue myeloperoxidase (MPO) activity and luminol and lucigenin CL levels in all groups in cirrhosis model formed by bile duct ligation (BDL) in rats (n = 6; mean ± SD)

	**Control**	**BDL**	**SPL1**	**SPL2**
MPO (U/g)	11.6 ± 2.9	39.1 ± 8.1^***^	24.0 ± 5.6^*, ++^	26.6 ± 9.6^**, +^
Luminol CL (rlu/mg)	3.6 ± 0.9	6.2 ± 0.8^**^	3.7 ± 1.5^++^	4.1 ± 0.9^+^
Lusigenin CL (rlu/mg)	2.9 ± 0.3	6.9 ± 2.5^**^	3.6 ± 1.3^++^	3.8 ± 1.0^+^

^*^p < 0.05, ^**^p < 0.01, ^***^p < 0.001 compared to the control group; ^+^*p <* 0.05, ^++^*p <* 0.01 compared to the BDL group.
